# One Health and Neglected Zoonotic Diseases

**DOI:** 10.3390/pathogens14050482

**Published:** 2025-05-15

**Authors:** Ricardo Pereira Igreja, Priscila Marques de Macedo, Maria Cristina Schneider

**Affiliations:** 1 Department of Infectious and Parasitic Diseases, Federal University of Rio de Janeiro, Rio de Janeiro 21940-590, Brazil; 2 Laboratório de Pesquisa Clínica em Dermatologia Infecciosa, Instituto Nacional de Infectologia Evandro Chagas, Fiocruz, Rio de Janeiro 21040-360, Brazil; priscila.marques@ini.fiocruz.br; 3 Department of International Health, Georgetown University, Washington, DC 20057, USA; mcs368@georgetown.edu; 4 Institute of Collective Health, Federal University of Rio de Janeiro, Rio de Janeiro 24220-900, Brazil

The vision that everything is connected in this world is not new. However, to respond to the current challenges that the world is facing, the integrated vision that humans, animals, and the environment are linked is more important than ever. Collaboration among multiple disciplines is crucial, and this approach is fundamental to understanding the One Health concept. A transdisciplinary definition of One Health describes animals, humans, and their shared settings or environments as linked and affected by the socioeconomic interests of humans and external pressures [1]. Zoonotic diseases, or zoonoses, are diseases shared between animals—including livestock, wildlife, and pets—and people. They can pose serious risks to both animal and human health and may have far-reaching impacts on economies and livelihoods. Zoonotic diseases are commonly spread at the human–animal–environment interface—where people and animals interact with each other in their shared environment. Zoonotic diseases can be food-borne, waterborne, or vector-borne, transmitted through direct contact with animals, or indirectly by fomites or environmental contamination [2]. A wide range of pathogens causes zoonotic diseases and could be classified as bacterial, viral, parasitic, fungal, rickettsial, chlamydial, mycoplasmal, or diseases caused by acellular non-viral pathogenic agents [3,4].

Zoonoses could have wide-ranging impacts on human, animal, and ecosystem health, and there are often complex social and political issues related to their assessment and management [5]. Many drivers could be described as related to the spillover of zoonotic diseases, such as agriculture and livestock production, the trade in wildlife, and wild meat consumption and climate change [1,6].

The movement of humans and animals carries with it the movement of pathogens. As such, the course of these journeys carries implications for the spread of zoonotic diseases. Across each of these supply chains are human–animal touchpoints, where disease can spillover to humans. Many captivity-raised animals are housed in large numbers in close confinement, enhancing the likelihood of disease spread. This unnatural mixing can give rise to new diseases, opening new doors for pathogens that might never have existed without our help, and creating networks and channels through which disease can spread from animals to humans and back again [7].

Neglected tropical diseases (NTDs) are ancient diseases of poverty that continue to cause hardship and harm to over one billion people worldwide, although some infections are so neglected that they do not even make it onto the official list of neglected diseases. They affect the most vulnerable—burdening people, families, and communities who are already marginalized and disadvantaged—despite being preventable and already having been successfully eliminated in many contexts [8]. Between 2012 and 2020, 31 countries eliminated at least one NTD. The number of people requiring interventions against NTDs has been reduced by 600 million since 2010 [9]. Many NTDs have a significant zoonotic component: Chagas disease, human African trypanosomiasis, leishmaniasis, lymphatic filariasis, onchocerciasis, dengue, and chikungunya are vector-borne; dracunculiasis, echinococcosis, foodborne trematodiases, rabies, schistosomiasis, and taeniasis/cysticercosis have animal reservoirs. Although snake bite is not infectious, it is a condition that mostly affects people living in poor communities; many are agricultural workers, hunter-gatherers, herders, fishermen, and indigenous people [10,11]. Human African trypanosomiasis (HAT) has been targeted by the WHO for elimination as a public health problem by 2030. Although five countries were recently declared free of HAT, rural populations engaged in farming, fishing, herding, or hunting have an increased risk of being bitten by tsetse flies [12].

Scholars that support the WHO’s list also recognize the importance of an expanded group of conditions that still qualify as NTDs due to evidence that they constitute chronic and debilitating conditions disproportionately affecting populations living in extreme poverty. In this expanded list, some zoonotic diseases such as leptospirosis, melioidosis, and Nipah virus infections are included [13]. Fungal infections, which are particularly neglected tropical diseases with zoonotic potential, are increasingly recognized within the One Health framework, which underscores the interconnectedness of human, animal, and environmental health. Human activities such as deforestation, urbanization, and climate change disrupt ecosystems, facilitating the spread of fungal pathogens like *Coccidioides*, *Paracoccidioides*, *Histoplasma*, and *Cryptococcus*, which are often transmitted through environmental or animal reservoirs. Fungal diseases are a major public health concern [14], and the epidemiology of many mycoses endemic to low-income countries has been significantly influenced by anthropogenic environmental changes [15]. Some of these diseases, such as cat-transmitted sporotrichosis, have a notable zoonotic component [16]. The One Health approach emphasizes integrated surveillance, zoonotic monitoring, and environmental management to address the growing threat of these infections, particularly in tropical and rural regions where human–animal–environment interactions are frequent. The control of stray animal populations, responsible pet ownership, the treatment of sick animals, and basic sanitation are critical measures to prevent the transmission of diseases like sporotrichosis to humans. While many of these fungal pathogens are included in the WHO’s fungal pathogens priority list, several significant fungal diseases have not been specifically recognized in the list of NTDs.

This is a crucial initiative, because many zoonotic diseases affecting vulnerable populations, such as leptospirosis and plague, do not always have the visibility to detect and prevent cases, nor those affected the tools to do so. These two bacterial diseases are excellent examples of the importance of the One Health approach, where rodents and certain environmental conditions play an important role [1]. It is estimated that one million cases of and around 60,000 deaths from leptospirosis occur worldwide annually, affecting, among others, poor farmers and slum dwellers in tropical regions [17]. Although leptospirosis is not considered a “tool-ready” disease for global initiatives, surveillance programs are not equipped with the tools for early detection and prevention, including human vaccines [18]. This disease remains imperceptible in many places, and is not included in the WHO’s roadmap for neglected tropical diseases nor considered a priority in many countries, mostly in Africa. The same number of deaths is estimated for rabies, 99% dog-mediated, with most occurring in Asia and Africa [19]. However, there are vaccines for people at risk of this ancient zoonotic disease and vaccines for prevention in dogs, and it is receiving more visibility as part of several initiatives where the One Health approach is recommended, such as “United Against Rabies” [20]. *Burkholderia pseudomallei* is a neglected tropical pathogen that is often misdiagnosed. Pig slaughterhouse workers and meat inspectors could be exposed to *B.pseudomallei* while handling infected pig carcasses [21].

Climate change affects disease distribution and transmission, putting poor communities in new places at risk. Changes in precipitation and temperature have enabled the endemic transmission of dengue, chikungunya, leishmaniasis, and schistosomiasis in parts of Central Europe and the USA [9]. Taking a One Health approach that recognizes the relationships between human, animal, and environmental health is key to sustainably addressing NTDs [8,20].

In an interconnected world that is undergoing considerable climate and environmental change, human, animal and environmental health are inextricably linked. While zoonotic risk cannot be eliminated, it can be reduced. Deforestation, urban expansion, climate change, and habitat destruction heighten the risk of infectious disease. Just as healthy animals are less susceptible to disease, research has shown that healthy, intact ecosystems spawn fewer disease outbreaks than degraded ones. Furthermore, biodiversity dilutes disease risk, acting as a natural buffer to the spread of pathogens. As the health of ecosystems suffers and more wild species are lost, we place ourselves at greater risk of zoonotic spillover [7].

As has been well known for 20 years, after Taylor et al.’s study [22], it was estimated that 61% of human pathogens worldwide were classified as zoonoses, as well as 75% of the emerging pathogens in the decade prior to the work. Subsequent studies proved that around 70% of infectious hazards that threaten public health are in the interface with animals [23,24]. There is no doubt about the importance of working in collaboration among disciplines and sectors, namely human health, animal health, and the environment.

Many initiatives related to zoonotic diseases recommend the One Health approach; among them is the Joint Action Plan for One Health launched by the quadripartite party in 2022 [25]. This joint One Health plan aims to create a framework for the integration of systems and capabilities that can collectively prevent, predict, detect, and respond to health threats. Ultimately, such an initiative seeks to improve the health of humans, animals, plants, and the environment while contributing to sustainable development, as recommended by the One Health High-Level Expert Panel (OHHLEP) [26]. This plan provides a framework for action and proposes activities that the four organizations (the FAO, WHO, UNEP, and WOAH, founded as the OIE) have developed together to sustainably scale up the One Health approach, aiming to drive the change and transformation needed to mitigate the impact of current and future health challenges at the human–animal–plant–environment interface at the regional, national, and global levels [25]. It complements and adds value to existing global and regional One Health initiatives, including by controlling and eliminating endemic zoonotic and neglected tropical and vector-borne diseases.

However, to address the much broader set of infectious disease risks associated with the Anthropocene, decision-makers will need to develop comprehensive strategies that include pathogen surveillance across species and ecosystems; conservation-based interventions to reduce human–animal contact and protect wildlife health; health system strengthening; and global improvements in epidemic preparedness and response [27].
